# Development of Thermal Spray Processes for Depositing Coatings on Thermoplastics

**DOI:** 10.1007/s11666-020-01147-x

**Published:** 2021-01-31

**Authors:** Kirsten Bobzin, Wolfgang Wietheger, Martin Andreas Knoch

**Affiliations:** Surface Engineering Institute, Aachen, Germany

**Keywords:** copper feedstock, substrate-coating interaction processing, high velocity wire-arc spray processing, PA6 substrate

## Abstract

Thermoplastics combine high freedom of design with economical mass production. Metallic coatings on thermoplastics enable power and signal transmission, shield sensitive parts inside of housings and can reduce the temperature in critical areas by functioning as a heat sink. The most used technical thermoplastics are polyamides (PA), while the described use cases are often realized using Cu. Consequently, several studies tried to apply copper coatings on PA substrates via thermal spraying; so far, this combination is only feasible using an interlayer. In this study, a new approach to metallize thermoplastics via thermal spraying based on validated state-of-the-art predictions of the thermoplastics’ material response at relevant temperatures and strain rates is presented. Using these predictions, high velocity wire-arc spraying was selected as coating process. Furthermore, the process parameters were adapted to realize a continuous coating while also roughening the substrate during coating deposition. The resulting Cu coating on PA6 had a sufficiently high coating adhesion for post-treatment by grinding. The adhesion is achieved by in situ roughening during the coating application. The results indicate that different process parameters for initial layer deposition and further coating buildup are required due to the low thermal stability of PA6.

## Introduction and State of the Art

Thermoplastics are widely used due to their intrinsic properties, i.e., high specific strength and modulus, suitability for mass production and a high degree of freedom regarding part design. Further properties include low thermal conductivity and excellent electric insulation (Ref 1). For example, the widely used thermoplastic PA6 has a short-time dielectric strength of V_B_ = 460 kVmm^−1^ and a thermal conductivity of *λ* = 0.23 Wm^−1^K^−1^ (Ref 2). These properties result from the covalent bonds found along the chains of the macromolecules and the secondary van der Waals bonds between the chains. Consequently, thermoplastics are excellent electrical and thermal insulators (Ref 1). The van der Waals bonds between chains unfortunately result in a low thermal stability of thermoplastics as well. Both the heat and electrical insulation can constitute a limitation to the design of thermoplastic parts. Therefore, various techniques have been employed to increase both properties locally, allowing the production of functional hybrid parts. Considering the thermal properties, heat sinks are often required to prevent overheating in the vicinity of heat sources like LEDs (Ref 3). By locally introducing a high electrical conductivity, conductor tracks can be realized in hybrid parts taking full advantage of multi-material design (Ref 4).

The combination of a metallic coating with a thermoplastic substrate can facilitate novel design opportunities, e.g., for hybrid parts with integrated electrical conductors or heat sinks, as well as an increased wear resistance. Consequently, various technologies for the metallization of thermoplastics have been developed. Industrially established is, for example, the coating deposition by physical vapor deposition (PVD) or electroless plating. Both techniques can produce high-quality coatings with typical coating thicknesses of *s* ≤ 10 µm. PVD coatings are deposited in a vacuum process, resulting in high invest and process costs. Electroless plating, on the other hand, requires additional pre-treatment steps. Both electroless plating and PVD coatings can further be used as pre-treatment for electroplating, enabling the deposition of thicker coatings at higher costs. Backmolding of metallic foils is also widely used with typical thicknesses of the metallic layer of 20 µm < *s* < 50 µm. Backmolding restricts part design to avoid warpage of the foils and requires additional process steps. (Ref 5-7)

Thermal spraying could offer a cost-effective solution for applying metallic coatings with a thickness of *s* > 100 µm on thermoplastics. The limited thermal and mechanical stability of thermoplastics does, however, pose a challenge for parameter development. In the past, numerous studies investigated possibilities to apply metallic thermal spray coatings on plastics. Most of these studies focused on thermosetting substrates (Ref 8), i.e., plastics that form a three-dimensional network of covalent bonds during the curing process. This network prevents cured thermosetting plastics from melting, resulting in a comparatively high strength and elastic modulus at elevated temperatures. These materials are brittle and cannot be formed after curing. Thermoplastics, on the other hand, can be melted and reshaped.

Unlike metals, thermoplastics exhibit a glass transition temperature *T*_g_, which is rather a temperature range. Above *T*_g_, the amorphous phase of thermoplastics has a high plastic deformability, while the material exhibits glassy characteristics below *T*_g_. Besides the change from brittle to ductile behavior, the ultimate strength *R*_b_ and the Young’s modulus *E* are substantially reduced above *T*_g_. The actual range of *T*_g_ can change significantly depending on the thermal history of the polymer, as well as the heating rate (Ref 9, 10). In case of semicrystalline thermoplastics, a certain degree of mechanical stability will be retained above *T*_g_ due to the presence of crystallites. These crystallites can be present in different phases, resulting in an influence of the thermal history on the mechanical properties (Ref 9). The mechanical properties of thermoplastics depend on the load velocity or frequency significantly as well (Ref 11). This effect is a result of the macromolecules’ inertia and thus intrinsic to thermoplastics. The relevant influences can be summed up as follows: Thermoplastics exhibit high specific moduli and strengths, especially in reinforced grades. Reinforcement is however only effective if the polymer matrix itself exhibits sufficient strength and moduli. Due to the low thermal stability of thermoplastics, they can only be used at low to moderate temperatures. Thermoplastics exhibit a softening temperature *T*_g_ and a melting temperature Tm, which represent a range, rather than a fixed point as is common for metals and ceramics. For both ranges, a significant difference between product data sheet values and actual properties during coating application must be assumed. Thermoplastics furthermore exhibit a low resistance to abrasive wear due to their low hardness. Thermoplastics like polytetrafluoroethylene (PTFE) are however used to reduce adhesive wear. The stiffness and strength of thermoplastic parts or substrates increase with increasing strain rate and decreasing temperature. These properties are influenced by the thermal history of the part as well.

**Fig. 1:**
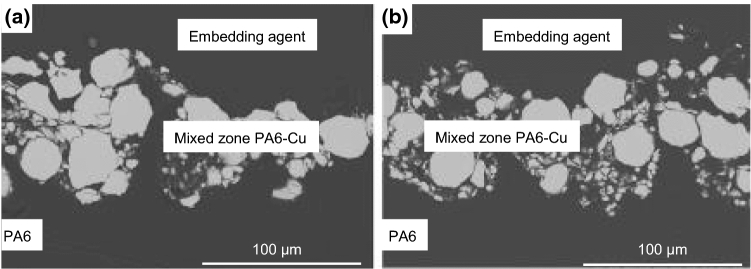
Particle embedding after cold spraying of Cu on PA6 substrates after one pass (a) and after three passes (b) after [14]

According to a review paper published in 2016 by Gonzalez et al. (Ref 8), studies on the metallization of polymers by thermal spraying have mostly focused on thermosetting substrates in the past. The studies considering thermoplastic substrates mostly used cold spraying (CS) to prevent excessive heat input into the substrates. As a rule of thumb, the state of the art can be summarized as follows: Metals with a low melting temperature, for example Sn, can be applied on various thermoplastics. If the melting point of the feedstock material is increased, substrates with higher thermal stability are required. If the thermal stability of the substrate is not sufficient, an interlayer of a coating material with a lower melting temperature can help to apply high-quality coatings. Feedstock materials like Al, Cu or Ti also require increased particle velocities and temperatures during CS. The increased temperature results in thermal softening of the substrate, which, in turn, prevents sufficient plastic deformation of the spray particles during impact. This often results in substrate erosion and the embedding of undeformed particles, see Fig. 1. (Ref 8, 12-14).

Cold-sprayed Cu coatings were only successfully applied on the high temperature thermoplastics polyether ether ketone (PEEK) and polyetherimide (PEI) without interlayer (Ref 12) and on the technical thermoplastics polyvinylchloride (PVC) (Ref 8, 13) and PA6 (Ref 14) using an interlayer. The direct application of a Cu coating on PA6 has so far not been feasible (Ref 8). Aside from CS, Ohmori et al. found a significant influence of the heat input into the substrate while depositing Cu on recycled polyethylene terephthalate (PET) via atmospheric plasma spraying (APS). Depending on whether the substrate surface reached its melting point or not, the Cu particles were embedded into the substrate creating a PET-Cu hybrid zone or a Cu coating was realized (Ref 15). More recently, the successful application of an Al coating on acrylonitrile butadiene styrene (ABS), polyethylene (PE) and PET using wire-arc spraying (WAS) were reported (Ref 3, 16).

None of the aforementioned studies considered recent advances in polymer science. In particular, models to predict the mechanical properties of thermoplastics at relevant load velocities and temperatures during thermal spraying are of interest in this context. These models are based on the works of Ree and Eyring (Ref 17, 18), who proposed that polymer solutions consist of different domains. Each domain exhibits a specific shear stiffness. During plastic deformation, the shear velocity is assumed constant within any individual shear plane, resulting in different shear stresses in the different domains. Furthermore, the deformation of each domain is independent of the other domains and the resulting deformation is the sum of the deformation of all individual domains. These theories have been validated for polymer solutions in the 1950s (Ref 17, 18). In recent years, sophisticated measurement equipment allowed for the adaptation validation of these theories for several thermoplastic melts, i.e., polycarbonate (PC) (Ref 11, 19), poly(methyl methacrylate) (PMMA) (Ref 11), polyamide-imide (PAI) (Ref 11), PEEK (Ref 20) polypropylene (PP) (Ref 21) and PA6 (Ref 22).

The goal of this study was to use these recent advances in polymer science to derive a novel interdisciplinary development method for the application of metallic coatings on thermoplastic substrates by thermal spraying (TS). The prediction of the substrate surface properties during coating application represents a vital aspect of this method. To validate the novel approach, PA6 substrates with 30% glass fiber reinforcement (PA6GF30) were coated with Cu without an interlayer for the first time.

## Interdisciplinary Development Method

**Fig. 2:**
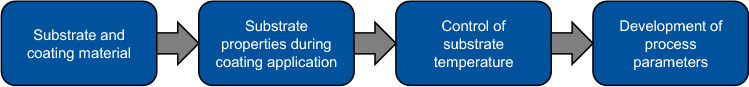
Novel interdisciplinary development method for the metallization of thermoplastics by thermal spraying

The novel development method is an extension of the traditional development method used in TS. As shown in Fig. 2, substrate and coating materials are selected first, based on technological and economical aspects, e.g., thermal stability, strength and price. Afterward, the substrate properties during coatings application are estimated based on the models introduced above. As a result of this step, acceptable substrate temperatures are derived and evaluated for the considered thermal spraying process variants. Based on the knowledge of acceptable substrate temperatures, measures to control the substrate surface temperature must be defined. Possible measures range from using colder process parameters over cooling by air jets to using a CO_2_ cooling system. Lastly, the process parameters are developed based on the assumptions made during the previous steps. The novel aspect of this method is the increased focus on the substrate properties as well as the adaption and/or development of analytical models and measurement setups to evaluate substrate heating and load velocity during coating application in future works.

To validate the applicability of the novel approach, PA6GF30-substrates were coated with Cu without the use on an interlayer. Cu coatings can, in general, be deposited with a wide range of TS process variants. The choice of process variant depends, in this case, mainly on the substrate properties and the heat input into the substrate.

It is well established that the properties of thermoplastic substrates are very sensitive to temperature and load velocity, i.e., momentum of the spray particles. The properties can furthermore be influences by moisture diffusing into the polymer. The considered substrate material consists of a PA6 matrix and 30 wt.% of discontinuous short glass fibers. At 23 °C, PA6 can absorb 9.5 wt.% water in saturation conditions according to ASTM D-570 and 2.7 wt.% water in 50% relative humidity conditions according to the same standard (Ref 2). The macromolecules of PA6 form hydrogen bridges between each other. The water molecules will predominately allocate themselves at these hydrogen bridges in the amorphous phase, acting as plasticizer. This increases the mobility of the macromolecules and consequently reduces *T*_g_ (Ref 6). Since the water absorption of PA6 is reversible, PA6 substrates should always be dried prior to metallization by TS to increase the mechanical stability of the substrate. To evaluate the substrate properties, the influence of water absorption will be neglected as its negative effects can easily be avoided.

As shown above, the limiting factor of thermoplastic substrates is the mechanical stability at elevated temperatures during coating deposition. While fiber reinforcement can enhance stiffness and strength of thermoplastic substrates, the reduction of both properties is caused by the matrix material. PA6 can form *α*-, at low cooling rates, or *γ*-crystallites, at elevated cooling rates. At temperatures below *T* = 125 °C, semicrystalline PA6 with α-crystallites is usually associated with a higher Young’s modulus (Ref 9). According to the product data sheet of the typical PA6 grade without reinforcement Akulon K122/F by DSM, a modulus of *E* = 2,3 GPa, a yield stress of *σ*_f_ = 80 MPa and a melting temperature of *T*_m_ = 220 °C can be assumed.

**Fig. 3:**
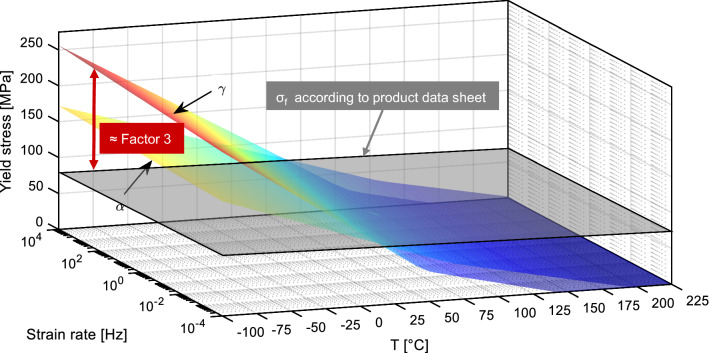
Approximation of σ_f_ of Akulon K122/F over the relevant temperature and strain rate during coating deposition

As discussed above, these values do not represent the real properties during coating deposition. Therefore, an approximation of the influence of temperature and load velocity, expressed as strain rate, on σ_f_ was performed. This approximation is based on the Ree–Eyring model mentioned above. Using a CO_2_ cooling system, substrate surface temperatures as low as *T* = − 78.5 °C can theoretically be achieved. Relevant strain rates Δε/Δt can be as high as Δ*ε*/Δ*t* = 10^5^ Hz in CS (Ref 23). The relevant substrate strain rate during impact is unknown. Considering the commonly assumed flattening time of spray particles between *t*_flattening_ = 50 ns in CS and *t*_flattening_ = 10 µs in flame spraying (Ref 24, 25), the strain rate in the current study is assumed to be at least one order of magnitude lower than the one reported in (Ref 23). Based on these considerations, the approximation using MATLAB 2019b was performed in a temperature range of − 100 °C < *T* < 225 °C and a strain rate range of 10^−4^ Hz < Δ*ε*/Δ*t* < 10^4^ Hz for *α*- and *γ*-containing semicrystalline PA6 using the material data of Akulon K122/F measured and validated in (Ref 26). Since only substrates in dry condition were used in this study, the corresponding formula from the same publication was used to visualize the data in the for this study relevant range. The results shown in Fig. 3 reveal that the temperature exhibits a more pronounced influence than the strain rate. Furthermore, the *γ*-crystallites can result in higher values for *σ*_f_, especially at lower temperatures and higher strain rates. At *T* = − 78.5 °C, the actual value of *σ*_f_ is roughly three times as high as the value found in the data sheet, highlighting the potential of sophisticated cooling systems.

**Fig. 4:**
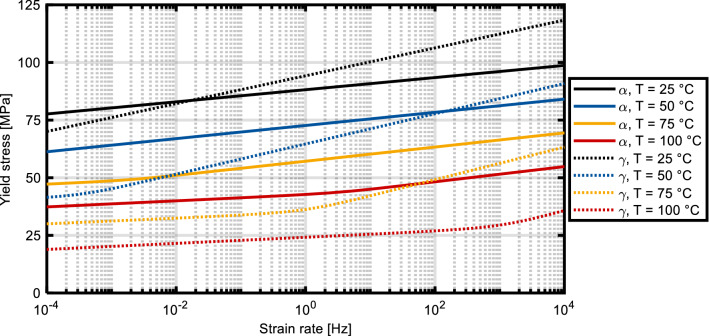
σ_f_ plotted over the strain rate for four discrete temperatures of α- and γ-PA6

The coating application in this study was performed without the aid of a substrate cooling system. The lowest surface temperature is therefore defined by the room temperature, assumed as *T* = 25 °C. Considering the significant reduction of *σ*_f_ above *T*_g_, *σ*_f_ was plotted over the strain rate for both crystallite types for *T* = 25 °C, *T* = 50 °C, *T* = 75 °C and *T* = 100 °C, see Fig. 4. At lower strain rates and temperature above *T* = 50 °C, the α-crystallites seem favorable. If a substrate surface temperature below 50 °C is achieved, e.g., by using a CO_2_ cooling system, *γ*-crystallites exhibit a higher yield stress, which can be beneficial to avoid substrate erosion. In this study, no cooling system was used and therefore the substrate surface temperature during initial contact between spray particle and substrate is assumed to exceed *T*_sub-surf_ = 50 °C. It should be noted that this temperate cannot be measured using conventional methods; IR sensors would only measure the temperature of the topmost layer, i.e., the Cu particles, while thermocouples would either measure a mixed temperature substrate and spray particle or, if embedded in a polymer, measure the temperature below the substrate surface. The development of suitable measurement techniques is subject of further research. Temperatures of *T* ≥ 75 °C result in low yield stresses, regardless of the present phases in PA6.

## Experimental Considerations

The goal of this study was twofold. Firstly, the direct deposition of Cu on PA6GF30 substrates was targeted. Realizing an in situ structuring of the substrate surface during coating deposition by taking advantage of the expected deformability constituted the secondary goal. In contrast to the particle embedding in (Ref 15), the deformation of a thin surface layer of the substrate was aimed at. If the temperature exceeds *T*_g_ significantly, substrate erosion is likely. Therefore, a substrate surface temperature during contact between spray particles and substrate of 50 °C ≤ *T*_sub-surf_ ≤ 85 °C was assumed as favorable. Considering the small temperature window and its proximity to the ambient temperature, the heat input into the substrate surface must be restricted. For this purpose, a low temperature process gas jet and small particles can be used. To avoid substrate erosion, the Cu particles should exhibit a higher deformability than the particles in (Ref 14). Avoiding substrate deformation completely is, however, not desirable as well with regard to the secondary goal. Therefore, the recently developed high velocity wire-arc spraying (HV-WAS) process was selected as coating process. The liquid state of the droplets helps to prevent substrate erosion despite the predicted yield stress reduction, see Fig. 4. Meanwhile, the heat input can be controlled by the wire feed rate, robot velocity and atomizing gas flux; the higher the gas flux is, the smaller the particles are (Ref 25).

The substrates were cut from an extruded PA6GF30 rod (Technoplast GmbH, Germany) with a diameter of Ø = 25 mm and a thickness of 10 mm. After cutting, the substrate surface was ground with #400 grit SiC paper to ensure a smooth surface and afterward stored in dry conditions for 1 week. Using x-ray diffraction, the presence of α-crystallites of the PA6 matrix was confirmed; *γ*-crystallite was not detected.

The coating application by HV-WAS was performed without substrate pre-treatment and without additional cooling using the GTV Precision Wire-Arc Spraying Unit (Präzisionslichtbogenanlage, GTV Verschleißschutz GmbH, Germany). The constant process parameters used during coating application are given in Table 1. The ambient temperature during the coating trials was above 30 °C but not recorded precisely. As stated above, the substrate surface temperature during contact constitutes the relevant temperature for this study. Since currently available equipment cannot capture this value with sufficient accuracy, no substrate temperature measurements were conducted to avoid confusion. One minute after coating deposition, all samples were slightly warmer than the ambient temperature.

**Table 1:** Constant process parameters during coating deposition

Parameter	Value
Wire	Cu (99,8%), Ø = 1,6 mm
Wire feed rate, g/min	60
Net power input, kW	3.2
Atomizing gas	N_2_
Robot velocity, mm/s	1000
Standoff distance, mm	200

To prevent substrate overheating, the spray gun was placed away from the substrate for 20 s after one or two passes, respectively. Furthermore, the gas flux was varied during in situ structuring and coating buildup. As starting point, 1500 SLPM were selected, which corresponds to a usual gas flux used in conventional wire-arc spraying. This parameter could not produce a continuous coating with sufficient adhesion. Consequently, coating delamination occurred during the deposition process and the coating partially fell off before a new layer buildup. An increase of the N_2_ flux to 1750 SLPM resulted in a thick and continuous coating.

**Fig. 5:**
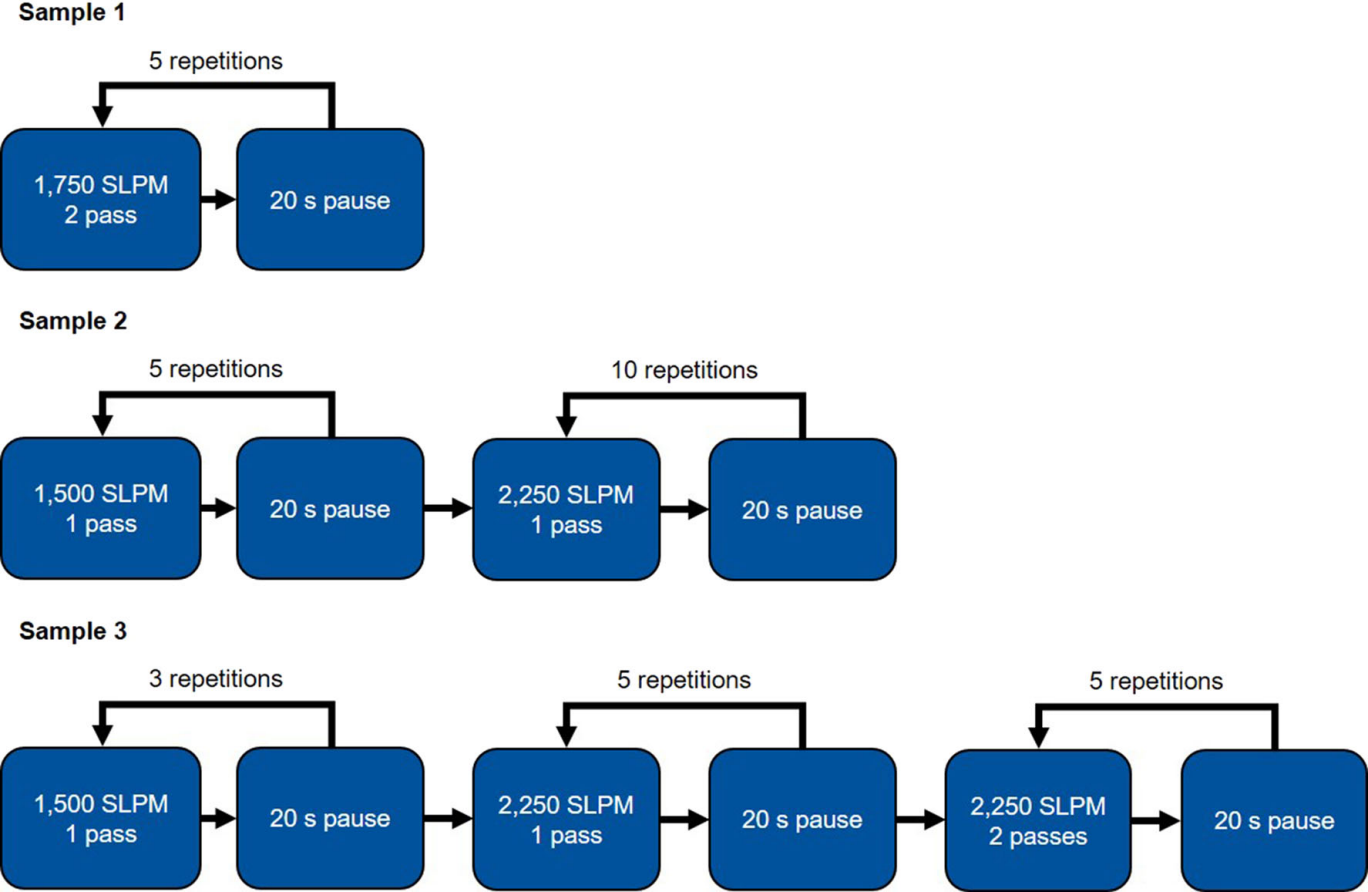
Coating strategies used to apply thick and continuous Cu coatings on PA6GF30 substrates

Based on these results, two further parameter sets were developed. Both use a N_2_ flux of 1500 SLPM for in situ structuring and a N_2_ flux of 2250 SLPM for coating buildup. The used coating strategies are displayed in Fig. 5. By switching from one process parameter to two process parameters in Sample 2, an increased local heat input is initially realized. This should increase the substrate deformation during the initial layer deposition. By limiting the number of passes to 1 between cooling cycles and to 5 in total for this structuring parameter, residual stresses in the coating are assumed to be reduced during this stage. Consequently, a thin coating was achieved. As mentioned above, this parameter was not suitable to deposit thick coatings due to delamination. Therefore, a coating buildup parameter was introduced. The higher gas flux should result in smaller, colder and faster particles. Both the decreased heat input and the increased kinetic energy of the particles increase the substrate’s yield stress. Sample 3 is based on Sample 2 but employs only three passes during structuring to reduce adverse effects on the coating adhesion. Afterward, the coating buildup parameter is used in two stages with decreased cooling pauses after the first five passes. The already deposited Cu coating has a significantly higher heat conductivity and thermal stability than the substrate. Therefore, the existing coating allows, in theory, for an increased heat input without damaging the substrate.

## Results and Discussion

**Fig. 6:**
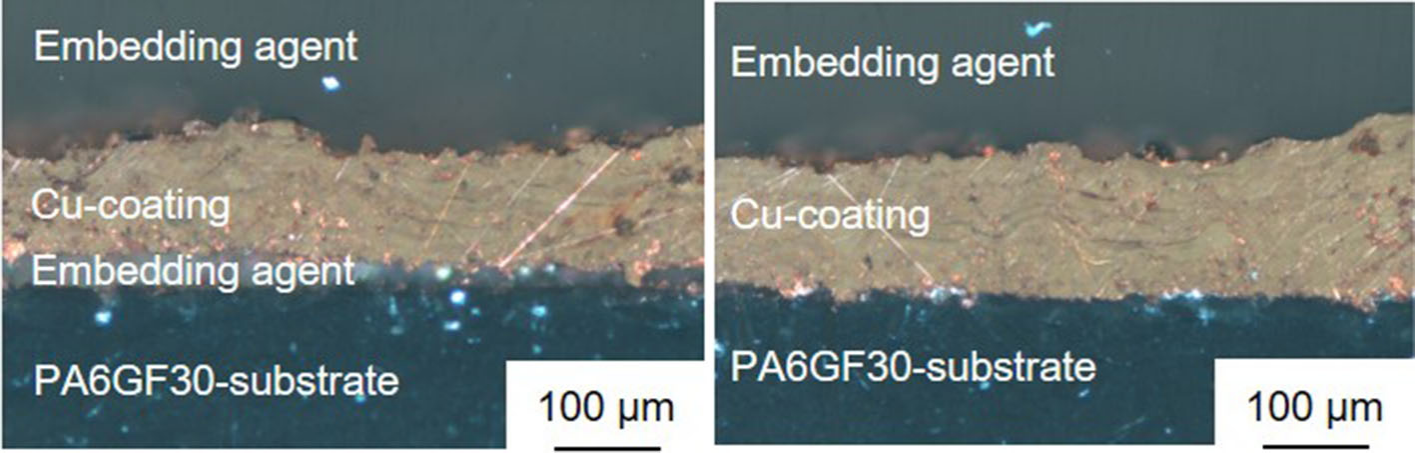
Polarization microscopy images of Sample 1 with local delamination (left) and good bonding between substrate and coating (right)

Increasing the gas flux from 1500 SLPM to 1750 SLPM enabled the application of a continuous and thick Cu coating. However, as indicated by the presence of the embedding agent between coating and substrate in Fig. 6 (left), local delamination occurred in the interface between Cu coating and the substrate. In contrast, some areas exhibited good bonding between coating and substrate, see Fig. 6 (right). It should be noted that no coating buildup was feasible if the wire feed was increased significantly. The increased wire feed results in higher net power input levels and thus a higher heat input into the substrate. It is also known that higher feed rates cause higher tensile stresses in the coating if all other parameters remain constant. The effects of the increased heat input on the in situ structuring and the increased stresses superpose. Therefore, it is not possible to determine which effect is dominant. It is likely that the increased heat input increased *T*_sub-surf_ above *T*_g_ which would correspond to a significant decrease of the substrate’s strength. The combination of decreased strength of the substrate and the existing tensile stress in the coating was assumed to be the reason for the delamination.

**Fig. 7:**
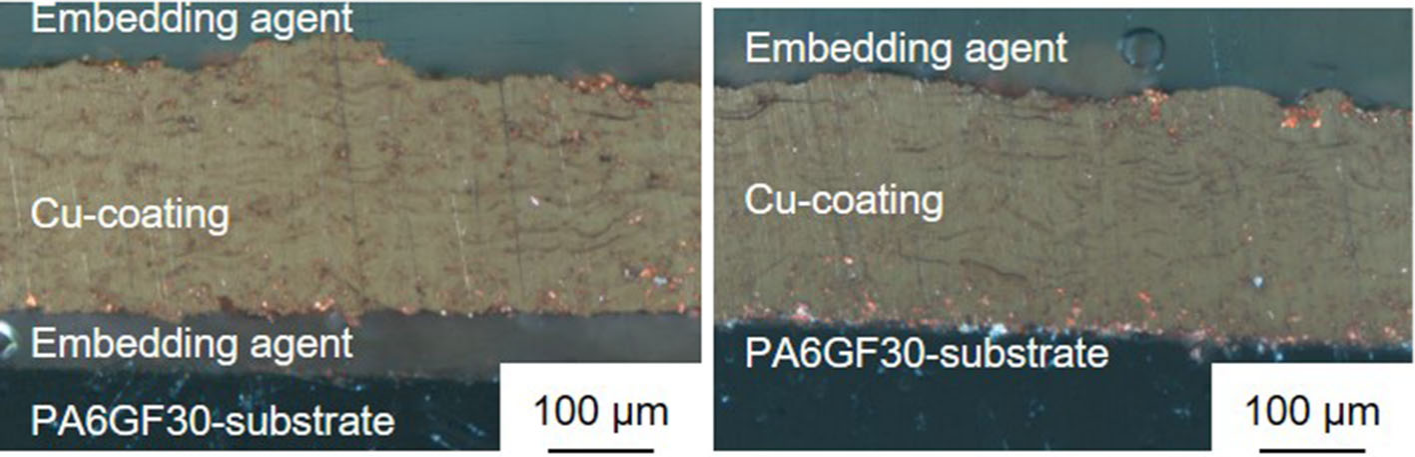
Polarization microscopy images of Sample 2 with local delamination (left) and good bonding between substrate and coating (right)

In case of Sample 2, coating deposition was also successful despite using a N_2_ flux of 1500 SLPM during in situ structuring of the substrate. As noted above, using 1500 SLPM during the entire process results in coating delamination. Despite the deposition of a continuous coating, the coating adhesion of Sample 2 was not sufficient, resulting in local delamination of the coating from the substrate, see Fig. 7. Although, this delamination was only observable in the cross sections, it highlights the importance of the local deformation of the substrate surface during in situ structuring. It should also be noted, that simply increasing the number of passes during in situ structuring resulted in coating delamination during the process, as well. Analogous to Sample 1, it is assumed, that the increased heat input into the substrate and higher tensile stresses cause this delamination.

**Fig. 8:**
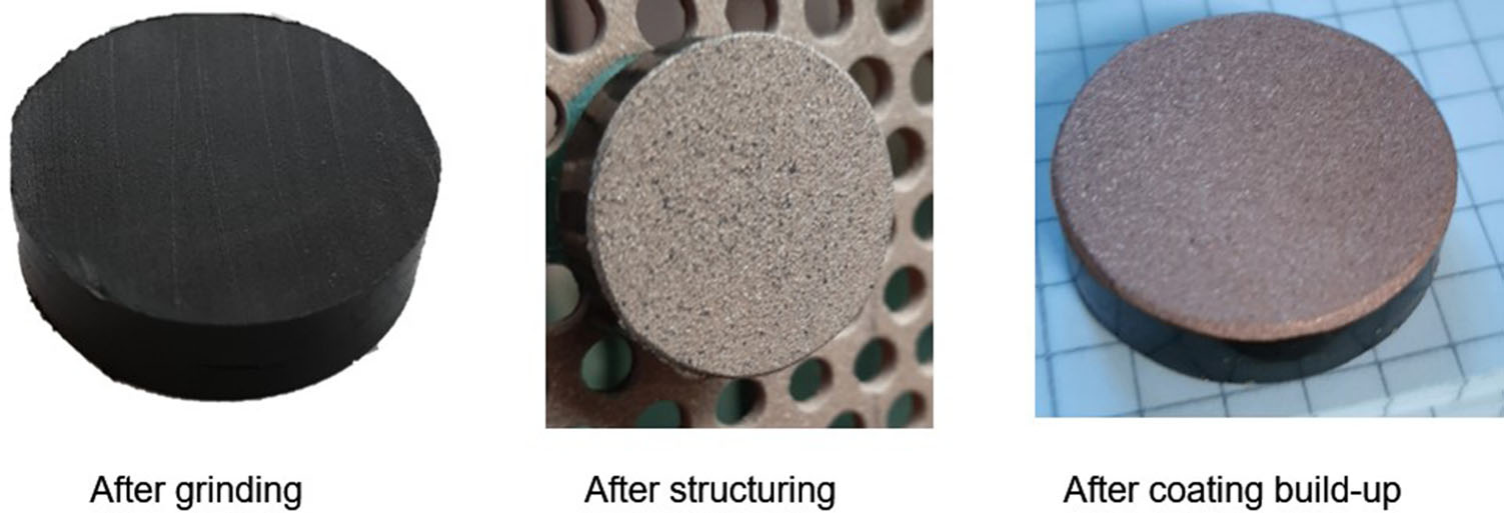
Images of Sample 3 before coating deposition, after the initial three passes and after the entire coating deposition process

**Fig. 9:**
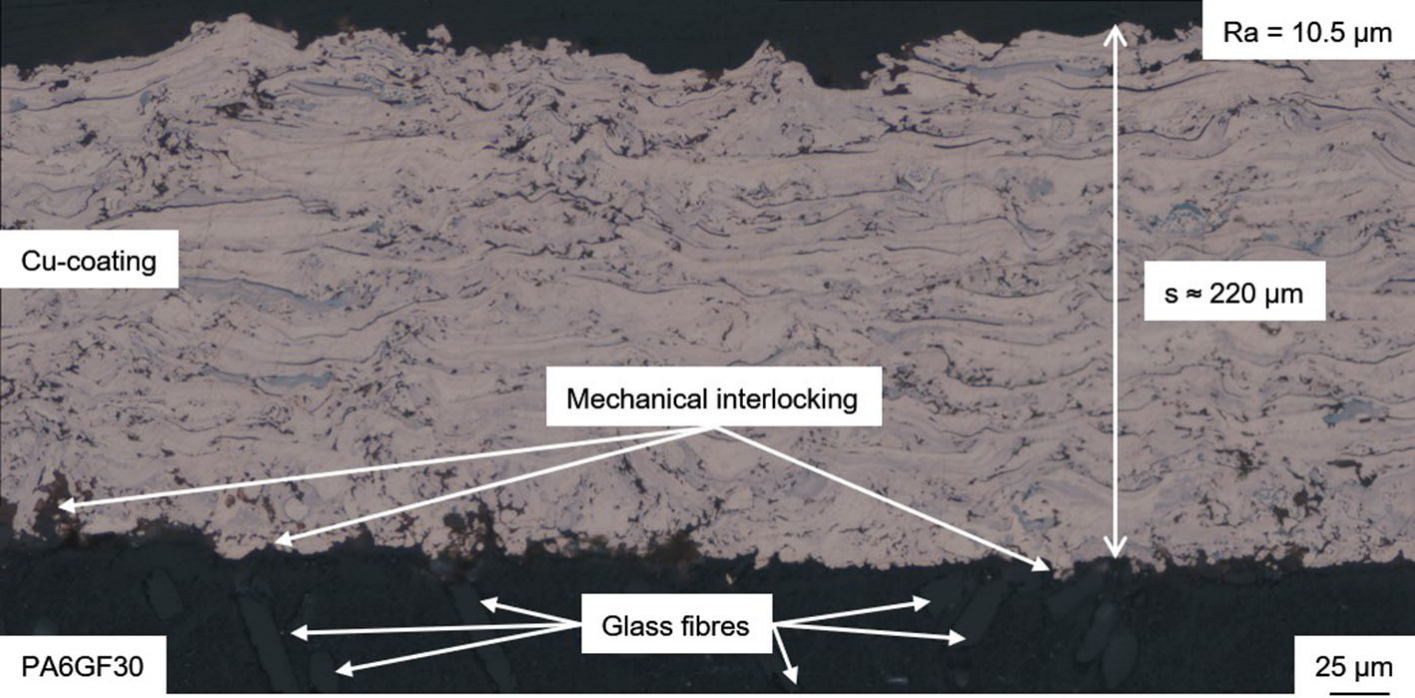
Bright field microscopy image of the Sample 3 coating microstructure

Considering Sample 3, the heat input, and tensile stresses of the first few layers, should be higher than in Sample 1. In Fig. 8, it is evident that the first three passes create a rougher surface. At this stage, first signs of delamination can also be observed, which ultimately lead to delamination if the coating thickness is increased using the same parameters. In the second and third step, the rough surface is filled with impacting particles interlocking with the rough PA6GF30/Cu surface created during the first three passes. The interface of Sample 1 shows distinct signs of local delamination, while no delamination was observed in Sample 3, see Fig. 9. Based on the good bonding, the parameters used for Sample 3 seem promising. It can therefore be stated that a lower heat input is not always beneficial. The heat input must rather be controlled precisely. In contrast, it must be assumed that lower tensile stresses are always beneficial to coating adhesion in the considered system.

The relatively small parameter variations between Samples 2 and 3 resulted in major differences during coating buildup and consequently the coating properties. In Sample 3, a continuous Cu coating was successfully applied on PA6GF30 for the first time without using an interlayer. The enhanced understanding of the substrate’s material response during coating deposition furthermore enabled an in situ roughening of the substrate.

This highlights that the in situ structuring is responsible for the comparatively good adhesion of Sample 3. The coating has a thickness of s ≈ 220 µm and exhibits a dense microstructure and a surface roughness of Ra = 10.5 µm as shown in Fig. 9. No delamination or defects, aside from glass fiber outbreaks due to the metallographic preparation, were observed in the interface between coating and substrate. The coating adhesion was high enough to enable mechanical post-treatment by grinding as well. After grinding with a #1200 grade SiC paper, the surface roughness was Ra = 0.5 µm; a lower roughness could be achieved by polishing the surface. Considering the target applications, electrical conductors or heat sinks, the coating adhesion can be considered sufficient. Since only Sample 3 exhibits sufficient adhesion, the other two parameter sets were not considered for further coating analysis.

**Fig. 10:**
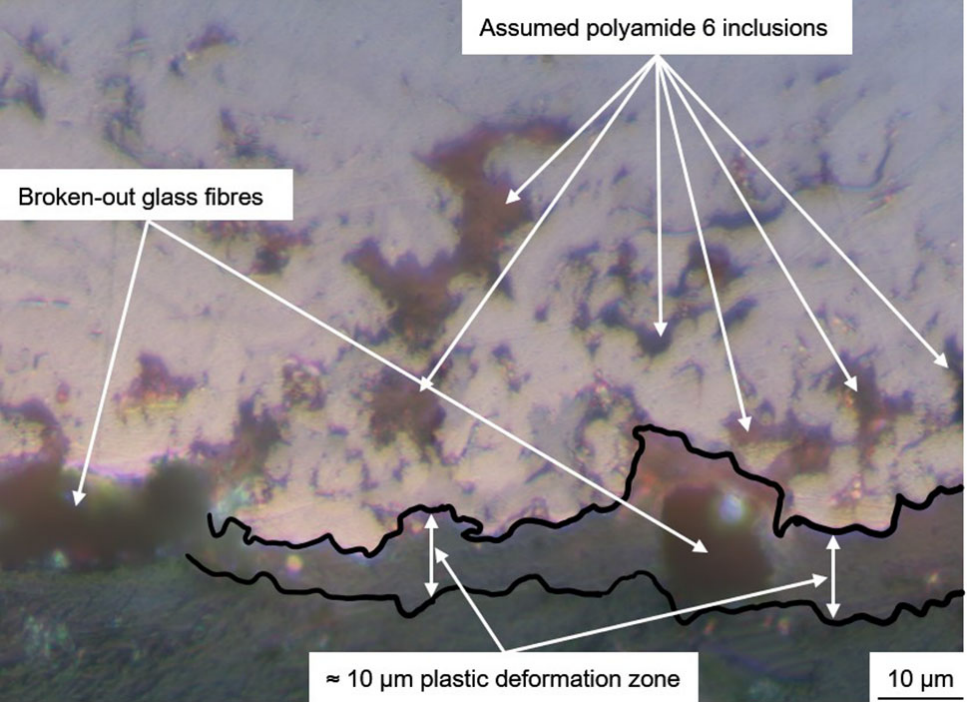
Polarization microscopy image of the substrate–coating interface and plastic deformation zone of Sample 3

Cu and PA6GF30 do not form chemical bonds. Therefore, the coating adhesion is based on mechanical interlocking due to the in situ roughening of the substrate. During the first passes, the low gas flux should have resulted in comparatively large and slow particles. The larger particles increase the local heat input into the substrate allowing for plastic deformation and the formation of a mixed zone analogous to (Ref 14). In contrast to the mixed zone in Fig. 1, the interface area of the coating in Fig. 10 contains significantly more Cu particles. Below the interface, a roughly 10 µm thick layer PA6 with a different structure can be found using polarization microscopy. This layer resembles extruded thermoplastics and assumedly formed due to the plastic deformation of the PA6 matrix during situ structuring. The low thickness of this plastic deformation layer on the substrate surface confirms the importance of the substrate surface temperature.

**Fig. 11:**
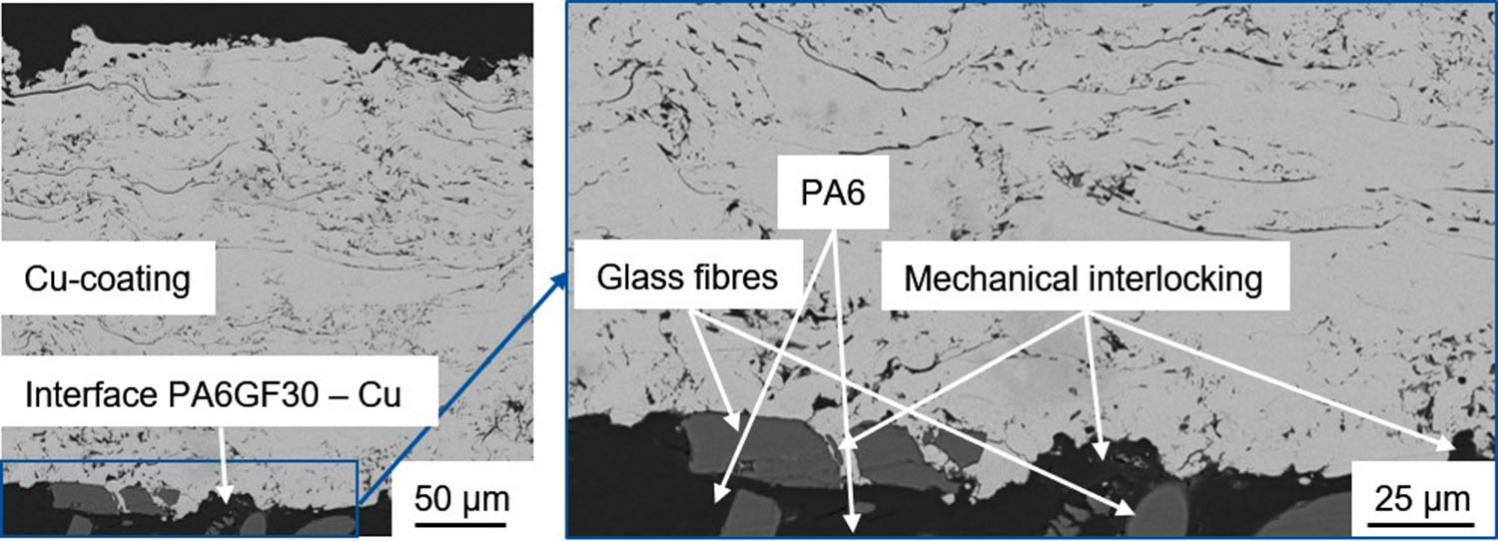
SEM images of Sample 3: overview of the coating morphology (left) and detail view of the interface between coating and substrate (right)

In Sample 3, some of the lamellae are partially oxidized despite the use of N_2_ as inert atomizing gas, see Fig. 9. For applications requiring thick Cu coatings with a low oxygen content, the use of active atomization gases, e.g., a mix of N_2_ and H_2_, could be used to limit particle oxidation (Ref 27, 28). Based on optical microscopy, particle oxidation was more pronounced than in previous studies which used conventional WAS with compressed air as atomizing gas to deposit Cu coatings (Ref 28). The SEM images shown in Fig. 11 reveal that the coating exhibits some porosity and the typical splat boundaries. The Cu coating itself appears to be rather homogenous. Therefore, the oxygen content in the coating can be assumed to be low, despite the change on optical properties. The interface between coating and substrate is well deformed, and both the polymer matrix and the glass fiber reinforcement contribute to mechanical interlocking.

**Fig. 12:**
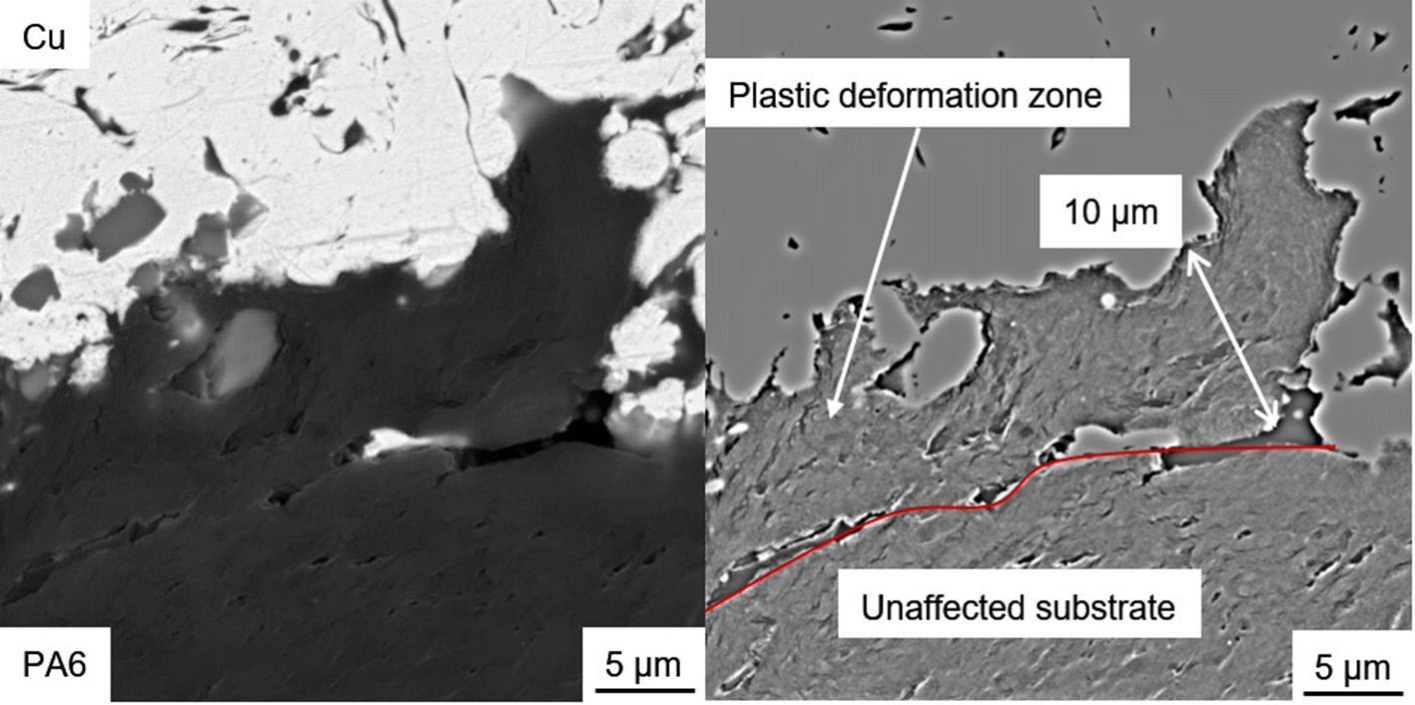
SEM image of the interface between coating and substrate of Sample 3 without post-processing (left) and after post-processing with a bandpass filter (right)

Considering the interface between coating and substrate, the in situ structuring during coating deposition is of special interest. To showcase the plastic deformation of the PA6 matrix, a representative SEM image of the interface is shown in Fig. 12. Compared to Fig. 11, this image was captured at a higher magnification and with increased brightness resulting in an overexposure of the Cu coating. The image without post-processing shows good mechanical interlocking between Cu coating and substrate. After adjusting brightness and contrast, to enhance the darker substrate areas, and applying a bandpass filter with 5% direction tolerance (imagej 1.52), to the image, two distinct zones can be observed in the substrate. The plastic deformation zone or extrusion zone was most likely formed during coating deposition and is a result of the desired in situ structuring. The lines in the right image of Fig. 12 follow roughly the outline of the Cu particles in the interface. Below this few µm thick zone, no signs of extrusion of PA6 matrix during coating deposition can be observed. The observed plastic deformation zone in Fig. 12 and 10 is identical. It should be noted that this zone is not visible in Fig. 9 and 11 since these images were captured with optical instead of polarization microscopy and at lower magnifications and with lower brightness settings, respectively.

## Conclusions and Outlook

In this study, a novel interdisciplinary development approach for the metallization of thermoplastic substrates by thermal spraying was presented. This approach takes advantage of advances in polymer science to predict the effective substrate properties during coating application and, thus, represents an extension of the state of the art. Using this approach, in combination with state-of-the-art coating equipment, a continuous, thick and dense Cu coating could be applied on PA6-based substrates for the first time without using an interlayer. Furthermore, the substrate pre-treatment could be reduced to storing the specimen in dry conditions prior to coating application. Mechanical interlocking between coating and substrate is achieved by in situ structuring the substrate during coating application. This requires precise control of the heat input into the substrate as well as of the particle impact. So far, small differences in the process parameters can result in coating delamination. The results indicate that initial layer deposition and coating buildup require different particle properties and therefore at least two different process parameters. Furthermore, the results confirm the importance of the substrate temperature previously reported in multiple studies. The substrate surface temperature seems to be vital for metallizing thermoplastics via thermal spraying. In further research, suitable systems to measure the temperature in the plastic deformation zone of the substrate should be developed to gain further insights. Such measurement systems would increase the use of the presented predictions immensely by providing the ability to define a target temperature range and check whether this temperature was achieved and how different cooling or heating systems can be utilized to achieve the desired substrate surface temperatures. Finally, it must be noted that the ambient temperature during the coating trials was above 30 °C. Considering the significant influence of small temperature variations of the substrate surface on σ_f_, see Fig. 4, different results might be observed if the coatings were applied at lower temperatures.
